# Transcriptionally imprinted glycomic signatures of acute myeloid leukemia

**DOI:** 10.1186/s13578-023-00981-0

**Published:** 2023-02-14

**Authors:** Constantin Blöchl, Di Wang, Oleg A. Mayboroda, Guinevere S. M. Lageveen-Kammeijer, Manfred Wuhrer

**Affiliations:** Center for Proteomics and Metabolomics, Leiden University Medical Center, Albinusdreef 2, 2333 ZA Leiden, The Netherlands

**Keywords:** *N/O*-glycans, Glycosphingolipids, Glycosyltransferases, Hematopoietic transcription factors, Primary blasts, AML cell lines

## Abstract

**Background:**

Acute myeloid leukemia (AML) is a genetically and phenotypically heterogeneous disease that has been suffering from stagnant survival curves for decades. In the endeavor toward improved diagnosis and treatment, cellular glycosylation has emerged as an interesting focus area in AML. While mechanistic insights are still limited, aberrant glycosylation may affect intracellular signaling pathways of AML blasts, their interactions within the microenvironment, and even promote chemoresistance. Here, we performed a meta-omics study to portray the glycomic landscape of AML, thereby screening for potential subtypes and responsible glyco-regulatory networks.

**Results:**

Initially, by integrating comprehensive *N*-, *O*-, and glycosphingolipid (GSL)-glycomics of AML cell lines with transcriptomics from public databases, we were able to pinpoint specific glycosyltransferases (GSTs) and upstream transcription factors (TFs) associated with glycan phenotypes. Intriguingly, subtypes M5 and M6, as classified by the French-American-British (FAB) system, emerged with distinct glycomic features such as high (sialyl) Lewis^x/a^ ((s)Le^x/a^) and high sialylation, respectively. Exploration of transcriptomics datasets of primary AML cells further substantiated and expanded our findings from cell lines as we observed similar gene expression patterns and regulatory networks that were identified to be involved in shaping AML glycan signatures.

**Conclusions:**

Taken together, our data suggest transcriptionally imprinted glycomic signatures of AML, reflecting their differentiation status and FAB classification. This study expands our insights into the emerging field of AML glycosylation and paves the way for studies of FAB class-associated glycan repertoires of AML blasts and their functional implications.

**Supplementary Information:**

The online version contains supplementary material available at 10.1186/s13578-023-00981-0.

## Introduction

Acute myeloid leukemia (AML) is a heterogenous hematological malignancy of the myeloid lineage, in which (epi)genetic variations result in neoplastic changes and clonal proliferation [1]. Eventually, immature blast cells present in the bone marrow, peripheral blood, and extramedullary tissues suppress normal hematopoiesis by their rapid growth and malignant signaling [2].

Glycosylation, a ubiquitous modification of biomolecules, is aberrantly controlled in many types of cancer and is increasingly recognized as one of the hallmarks thereof [3]. Next to the study of individual glycans, investigating glycan traits or epitopes represents a particularly insightful way to assess the altered glycobiology of different cancer entities [3, 4]. In hematological malignancies, i.e., leukemias and lymphomas, glycomic changes have been reported for all major classes, namely *N*-glycans, *O*-glycans, glycosaminoglycans, and glycosphingolipids (GSLs) [5]. Although there are currently only limited insights available for AML, cellular glycosylation seems to play a particular role in this malignancy [6]. Aberrant glycosylation may fundamentally alter pathological intracellular signaling in AML: i. NOTCH signaling, an important pathway with both oncogenic and tumor-suppressive potential in AML [7], changes levels of glycosyltransferases (GSTs) that are involved in *O*-fucosylation and *O*-glucosylation, which is eventually resulting in a positive feedback loop [8–11]; ii. correct glycosylation of the FMS-related tyrosine kinase 3 (*FLT3*) is essential for its tumorigenic effects in AML and altering its *N*- and *O*-glycans may be harnessed as a potential therapeutic approach [12].

Recently, two studies by our group investigated glycomic signatures of a broad panel of AML cell lines [13, 14]: both protein (*N*- and *O*-linked) [13] and GSL glycosylation [14] revealed a surprisingly diverse glycosylation across these cell line models. In addition, several glycan traits were found to be associated with AML subtypes as classified by the French-American-British (FAB) system [15]. In contrast to the WHO system [16, 17], the FAB classification does not take into account recurrent genetic aberrations but assigns AML into eight subtypes (M0-M7) based on cellular morphology, cytochemical characteristics, and cellular differentiation [15]. The generally low differentiation level of AML blasts represents a key feature of the disease and induction of differentiation has been extensively researched as a treatment option [18]. Notably, the exogenously induced differentiation of AML blasts and its impact on cellular glycosylation has been explored in earlier studies: Delannoy et al*.* reported drastic changes in the levels of sialylated GSLs paired with a significant induction of GM3 (NeuAcα2-3Galβ1-4Glcβ1-Cer) after the differentiation of the AML cell line THP-1 along a macrophage lineage [19]. In addition, sialyl Lewis^x^ (sLe^x^) expression on the surface of differentiated cells was reduced, most likely being *N*-glycan associated. In another report, cellular differentiation could be induced by the exogenous addition of GM3 in the HL-60 AML cell line [20]. In the same cell line model, GM3 biosynthesis could be induced by phorbol 12-myristate 13-acetate (PMA) [21], a well-known inducer of differentiation, and was linked to the expression of GST *ST3GAL5*.

In addition, cell surface glycosylation of AML blasts may fundamentally shape the interactions with cells of their microenvironment. On the one hand, glycoforms of CD82 alter the attachment to cells of the bone marrow with potential implications in chemoresistance [22–24]. On the other hand, the sLe^x/a^ epitope on the blasts’ surface facilitates binding to E-selectin expressed by endothelial cells in the vascular niche of the bone marrow. Through activation of multiple pro-survival signaling pathways, this sLe^x/a^ – E-selectin axis has been shown to confer chemoresistance in AML [25–28]. In accordance with these mechanistic studies, the increased expression of GSTs *ST3GAL4* and *FUT7,* both linked to the biosynthesis of sLe^x^, were associated with poorer survival of AML patients [29]. With these first insights at hand, there is great hope that the malignancy-associated expression of sLe^x/a^ in AML can be exploited to improve the outcome of this disease. One strategy employs the glycomimetic drug uproleselan (GMI-1271), which is a specific E-selectin antagonist [30]. After showing promising results in a phase I/II study enrolling relapsed or refractory AML patients, currently a phase III trial is ongoing (NCT03616470) [31].

Although to date there is limited knowledge available about the role of glycosylation in AML, an increasing number of studies report its involvement in malignant signaling, chemoresistance, and stratification of the disease. Moreover, they outline how these findings may be harnessed to increase the prognosis of AML and reduce the amount of relapsed or refractory cases, which pose a major obstacle in its treatment. To broaden our understanding of the glycosylation landscape of AML, we conducted an integrated glycomics and transcriptomics study relying exclusively on publicly available datasets to map protein- and lipid-linked glycosylation in AML cell lines and primary cells. Thereby, we intend to shed light onto the regulation and stratification of cellular glycosylation in AML and its subtypes and explore the clinical relevance of these findings.

## Materials and methods

### Data collection

Glycomics data from AML cell lines were obtained from GlycoPOST [32] and the respective publications: GPST000214 (*N*- and *O*-glycomics) [13] and GPST000238 (GSL-glycomics) [14]. Where available, GlyTouCan identifiers (https://glytoucan.org/) were assigned to the identified structures [33]. AML cell line transcriptomics data was obtained from the depmap portal (Expression 22Q2 Public; Broad Institute, Cambridge, MA, USA) [34]. Glycomics and transcriptomics data could be retrieved from the following 19 cell lines (information has been retrieved from the Cellosaurus database [35] listing resource identifiers (RRIDs)): AML-193 (RRID:CVCL_1071), EoL-1 (RRID:CVCL_0258), HEL (RRID:CVCL_0001), HEL 92.1.7 (RRID:CVCL_2481), HL-60 (RRID:CVCL_0002), Kasumi-1 (RRID:CVCL_0589), KG-1 (RRID:CVCL_0374), KG-1a (RRID:CVCL_1824), M-07e (RRID:CVCL_2106), ME-1 [Human leukemia] (RRID:CVCL_2110), ML-1 [Human leukemia] (RRID:CVCL_0436), MOLM-13 (RRID:CVCL_2119), MV4-11 (RRID:CVCL_0064), NB4 (RRID:CVCL_0005), OCI-AML-3 (RRID:CVCL_1844), PLB-985 (RRID:CVCL_2162), THP-1 (RRID:CVCL_0006), U-937 (RRID:CVCL_0007), and TF-1 (RRID:CVCL_0559). Comparison of gene expression patterns and correlation analysis in primary AML cells was conducted on a large pre-compiled next-generation sequencing dataset (GSE122515) [36] available from the Gene Expression Omnibus (GEO) [37]. FAB-classified data were obtained from the GSE12417 training and test set [38] and the GSE37642 dataset [39] retrieved from GEO [37].

### Data analysis

For the analysis of AML cell line glycomics data, cell lines were only included if information was available for all three glycan classes. Quantitative values for individual glycans were translated into glycan traits as specified in the Additional file 2: Tables S1, S2, and S3 and summarized in Additional file 2: Table S4. Principal component analysis (PCA) was performed in SIMCA 13.0.3.0. To avoid a bias in the weight of the individual datasets, the data was scaled to unit variances and approximately the same amount of features were used per dataset. Spider plots were created after *z*-transformation taking into account all investigated cell lines. Correlation analysis of glycan traits with GSTs and TFs in cell lines was performed using Spearman correlation.

Matrix to matrix correlation of GSTs/TFs expression in cell lines and primary cells (GSE122515 [36]) was calculated using the RV2 coefficient according to Smilde et al. [40].

For analysis of FAB-grouped data of primary cells, the GSE12417 training and test set [38], and the GSE37642 dataset [39] were compiled and subsequently normalized by limma [41]. The data subsets measured on the Affymetrix Human Genome U133A array (referred to as the GPL96 platform within GEO) were used for all evaluations. This microarray approach allows determination of gene expression levels of a predefined set of around 39,000 transcripts. If multiple Affymetrix probe sets were available for a single gene, probe sets were chosen manually with the help of the USCS genome browser [42] aiming at the best overlap for cDNA exonic sequences.

Data analysis and visualization were performed in GraphPad Prism 9.3.1. and within the “R” environment (version 4.0.5) including packages “tidyverse”, “corrplot”, “limma”, and “GEOquery”.

## Results

**Fig. 1:**
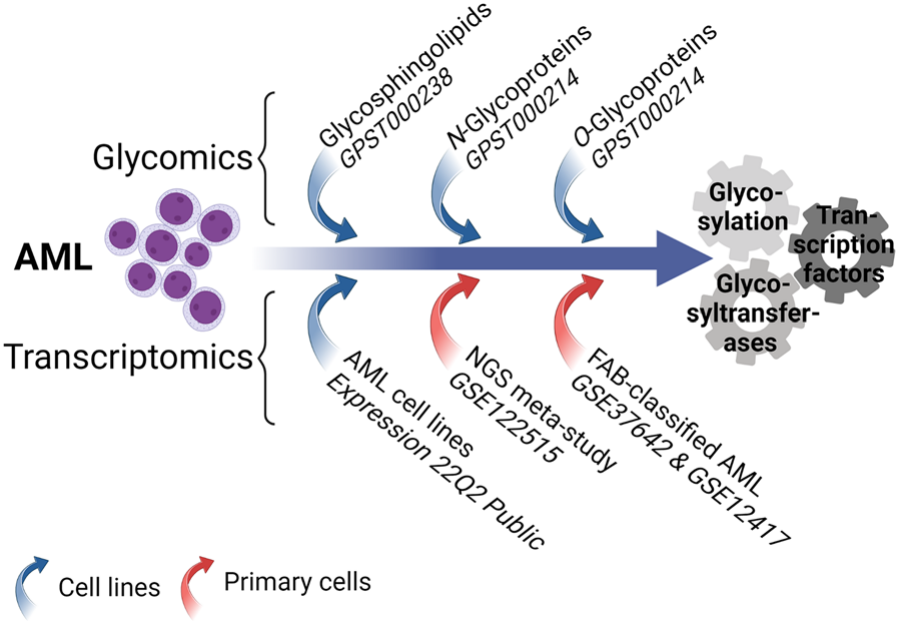
Overview of the conducted study. AML glycosylation was explored on the level of glycomics (GPST datasets) and transcriptomics (GSE and DepMap datasets). Based on the depicted datasets originating from cell lines and primary cells we sought to explore cellular glycosylation, involved GSTs, and responsible TFs

In this study, we set out to expand our understanding of protein and lipid glycosylation and their regulation in AML to obtain insights that may affect the development of diagnostic or therapeutic approaches. To this end, we retrieved glycomics and transcriptomics data from multiple studies dealing with both cell line models and primary blasts (Fig. 1). Employing the well-defined and comprehensively assessed cell lines as a stepping stone, we intended to define glycosylation of AML blasts as well as its transcriptional regulation on the level of GSTs and TFs.

### Integration of glycan classes in AML cell lines and their association with the FAB classification

**Fig. 2:**
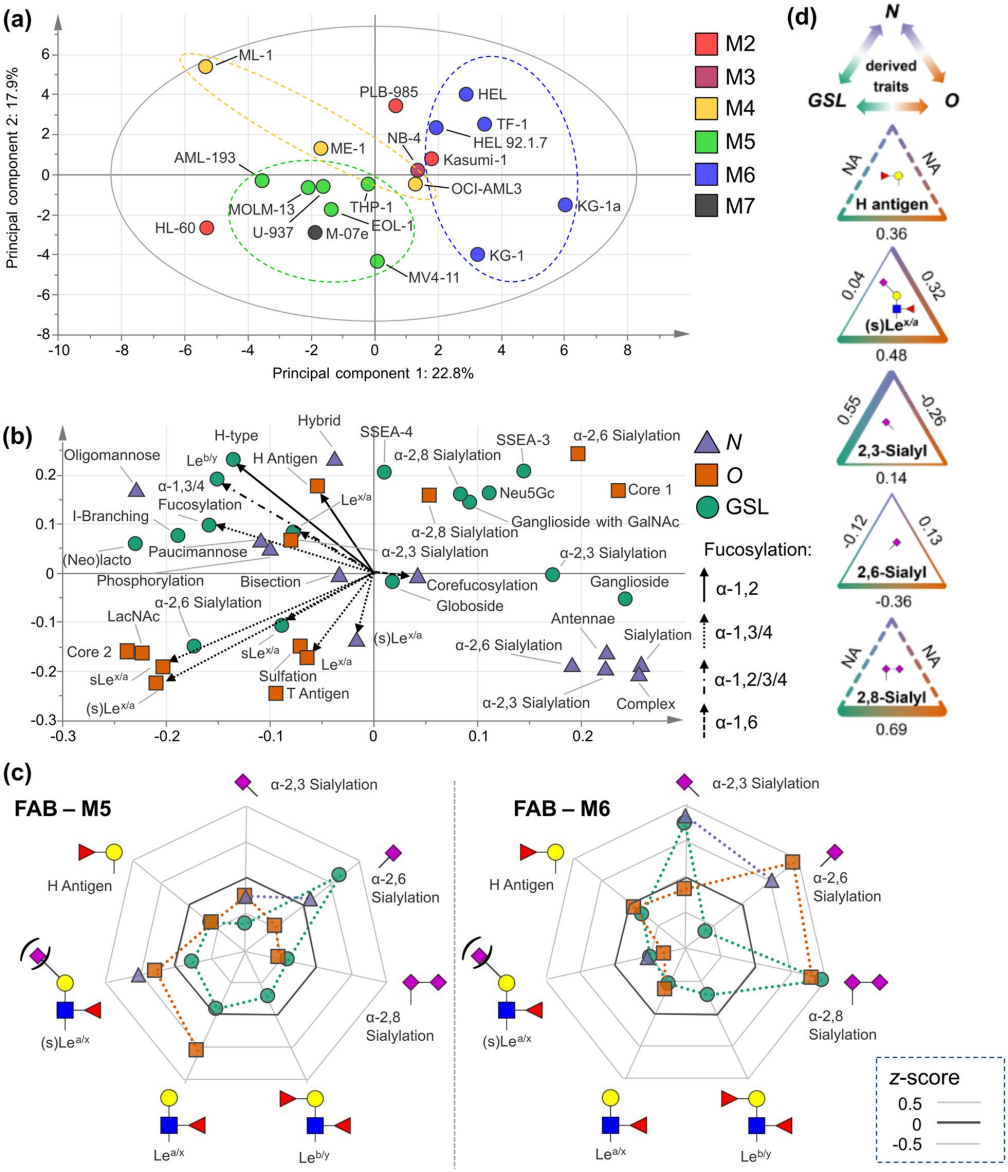
Glycomic overview of various AML cell lines. **a** PCA of glycosylation features derived from glycomics data of 19 AML cell lines. Individual cell lines are annotated and colored by their FAB classifications as assigned earlier [13]. **b** The associated score plot depicts considered glycan features, which are linked to their respective glycan class (*N*-, *O*-, and GSL) by color (purple, orange, and green) and symbol (triangle, square, and circle). In addition, arrows indicate features that are linked to a specific type of fucosylation. **c** Radar plots are showing the differences in glycosylation features between AML classes M5 and M6. Again, these features are subdivided into their respective classes based on color and symbols. Data on all AML cell lines were z-transformed prior to visualizing differences between FAB classes in these radar plots. **d** Spearman correlation of selected glycosylation features between the different glycan classes. Thick connective lines indicate a good correlation whereas thin connective lines show less correlation. Correlation values are depicted

The bioinformatic evaluation of the AML glycomic landscape was initiated by relying on cell line models, as the most detailed glycomics and transcriptomics data were available for these cells. Recently, we reported on the in-depth glycan characterization of numerous AML cell lines, i.e., *N*-, *O*-, and GSL-glycosylation [13, 14]. To obtain a holistic picture of cellular glycosylation of AML cell lines, to explore cooperative trends, and to determine unique features, we integrated available data [13, 14] on these three glycan classes. To this end, glycans were grouped into glycosylation features as specified in Additional file 2: Table S1 (*N*-glycans), 2 (*O*-glycans), and 3 (GSL-glycans). The compiled quantitative information is available in theses supporting tables. The relative abundances of the different glycomic features across 19 AML cell lines were explored by PCA, which pointed towards clustering of cell lines by their FAB classification as visualized in the score plot (Fig. 2a). In particular, cell lines from the M5 class (acute monocytic leukemia cell lines: AML-193, MOLM-13, U-937, THP-1, EOL-1, and MV4-11) showed an apparent grouping driven by relatively high expression of (s)Le^x/a^ across all three glycan classes (Fig. 2b). In addition, some class-specific glycan features contributed to the clustering of M5 cell lines, namely *O*-glycan associated core 2 and sulfation, *N*-acetyllactosamine (LacNAc), T antigen, as well as GSL-glycan-associated α-2,6 sialylation. Besides, the cell line M-07e, which is derived from the M-07 (RRID:CVCL_D630) cell line andbelongs to the M7 class (acute megakaryoblastic leukemia), showed similar glycosylation features as the M5 cell lines. However, as this was the only M7 cell line included, it remains unclear as to whether this is a common pattern of M7 cells or unique for this particular cell line. Cell lines of the M6 subtype (acute erythroid leukemia: HEL, HEL 92.1.7, TF-1, KG-1a, and KG-1) clustered and were separated from the M5 cell lines in the score plot (Fig. 2a). Above all, different forms of sialylation were drivers of the clustering of these M6 cell lines, i.e., *O*- and GSL-glycan-associated α-2,8 sialylation, Neu5Gc on GSLs, α-2,3/6 sialylation on *N*-glycans, and α-2,6 sialylation on *O*-glycans, (Fig. 2b). The two sister M6 cell lines (KG-1a and KG-1) clustered particularly close due to the high expression of (α-2,3/6) sialylation on *N*-glycans and their high antennarity as well as ganglioside GSL-glycans. To further facilitate the comparison of M5 and M6 subtypes, we visualized the *z*-transformed data of the most important glycan features (Additional file 2: Table S5) in two radar plots (Fig. 2c) revealing differences in (s)Le^x/a^ expression and various types of sialylation in a glycan class-specific manner.

While the three cell lines of the M4 class (acute myelomonocytic leukemia) showed less clear grouping, they were in part characterized by H antigen expression on *O-* and GSL-glycans. For the M2 subtype, three cell lines were typed with no clear common glycan signatures becoming apparent.

To investigate whether the same glycan features show distinct expression across the investigated glycan classes, we performed a correlation analysis thereof (Fig. 2d). Common features across glycan classes included (s)Le^x/a^ and α-2,3/6 sialylation, whereas H antigen and α-2,8 sialylation were only found on *O*- and GSL-glycans. Between *O*- and GSL-glycans, positive correlations were found for H antigens (r = 0.36), (s)Le^x/a^ (r = 0.48), and α-2,8 sialylation (r = 0.69). In contrast, weak negative correlations were found for α-2,6 sialylation on GSL-glycans compared to *N-* and *O*-glycans (r = − 0.12 and − 0.36, respectively). Between *N*- and GSL-glycans, α-2,3 sialylation showed a positive correlation (r = 0.55).

### Exploring the correlation of glycomic features and GST expression in AML cell lines

**Fig. 3:**
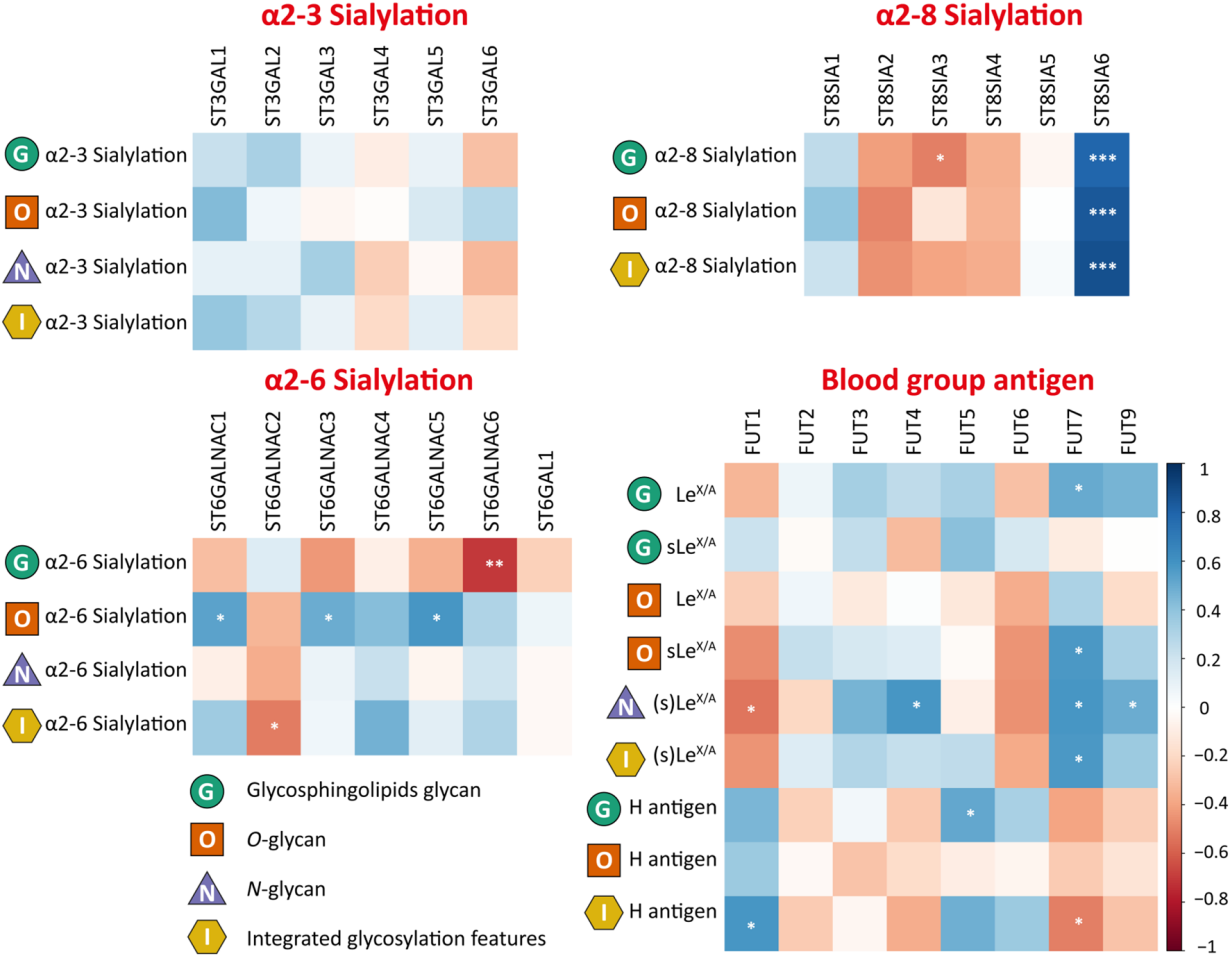
Correlation of glycomic features on *N*-, *O*-, and GSL-glycans with GST expression. Coefficients were obtained by Spearman correlation and are colored as indicated in the right key bar. Integrated glycosylation traits were obtained by summing up values of class-specific glycosylation traits. Significant values are marked with * (p ≤ 0.05), ** (p ≤ 0.01), and *** (p ≤ 0.001). Correlation coefficients and p-values are listed in the Additional file 2: Table S7

Biosynthesis of glycans and resulting cellular glycomic features are shaped by GSTs. To explore associations of specific GSTs and related glycosylation features, we performed a Spearman correlation analysis between glycomics and transcriptomics data (Additional file 2: Tables S5, S6, and S7). Previously, we investigated the associations of GSTs with glycomic features specific to individual glycan classes [13, 14]. For this study, we focused on glycan traits shared between the three glycan classes, i.e., α-2,3/6/8 sialylation and blood group antigens (Fig. 3). For this purpose, glycan traits, e.g., sLe^x/a^ or α-2,3 sialylation, were calculated by summing up the relative abundances of glycans that carry the respective trait. If multiple of these epitopes were present on one glycan, the relative abundance of this glycan was multiplied by the number of epitopes identified. Glycan traits were integrated across glycan classes, i.e., *N*-, *O*-, and GSL-glycans, by summing up their values throughout the three classes in order to observe the impact of specific GSTs on the global glycomic phenotype of AML blasts (Additional file 2: Tables S1, S2, S3, S4, and S5). With respect to α-2,3 sialylation, no significant correlations were found for *ST3GALs* in the different glycan classes. Of note, *ST3GAL1,* encoding a GST involved in terminal sialylation of glycoproteins and glycolipids, showed a trend toward a positive correlation with α-2,3 sialylation on both *O-*glycans and GSL-glycans. Similarly, *ST3GAL2*, which encodes a GST thought to be specifically responsible for the production of terminal sialylation of glycolipids, positively correlated with α-2,3 sialylation on GSL-glycans. Next, correlations were investigated for GSTs responsible for the formation of α-2,6 sialylation. Strong correlations were found between *ST6GALNAC1/3* and α-2,6 sialylation on *O-*glycans. Despite its preferential activity on glycolipids [43, 44], *ST6GALNAC6* expression negatively correlated with GSL-associated α-2,6 sialylation*.* As for α-2,8 sialylation, *ST8SIA6* significantly correlated with this trait on *O*- and GSL-glycans, whereas this feature was absent on *N*-glycans.

Finally, blood group antigens were assessed. Specifically, *FUT1-7* and *FUT9* were included in the analysis, whereas *FUT8* was excluded as it is not involved in blood group antigen synthesis. *FUT1*, which is responsible for catalyzing the transfer of a fucose to a terminal galactose residue of glycoconjugates in α-1,2 linkage to form H antigens, showed a significantly positive correlation with global H antigen expression, whereas its expression on *O*- and GSL-glycans individually did not reach the significance threshold. Concerning (s)Le^x/a^ expression, the strongest positive correlations were found for *FUT7* and *N*- and *O*-glycosylation. Global expression of (s)Le^x/a^ on *N-, O*-, and GSL-glycans, correlated significantly positively with *FUT7* expression. In addition, *FUT4* and *FUT9* appeared to be significantly associate with (s)Le^x/a^ expression on *N*-glycans. Interestingly, overall (s)Le^x/a^ expression seems to negatively correlate with *FUT1* and *FUT6* expression.

### Integrated glycosylation features reveal key transcription factors in AML cell lines

**Fig. 4:**
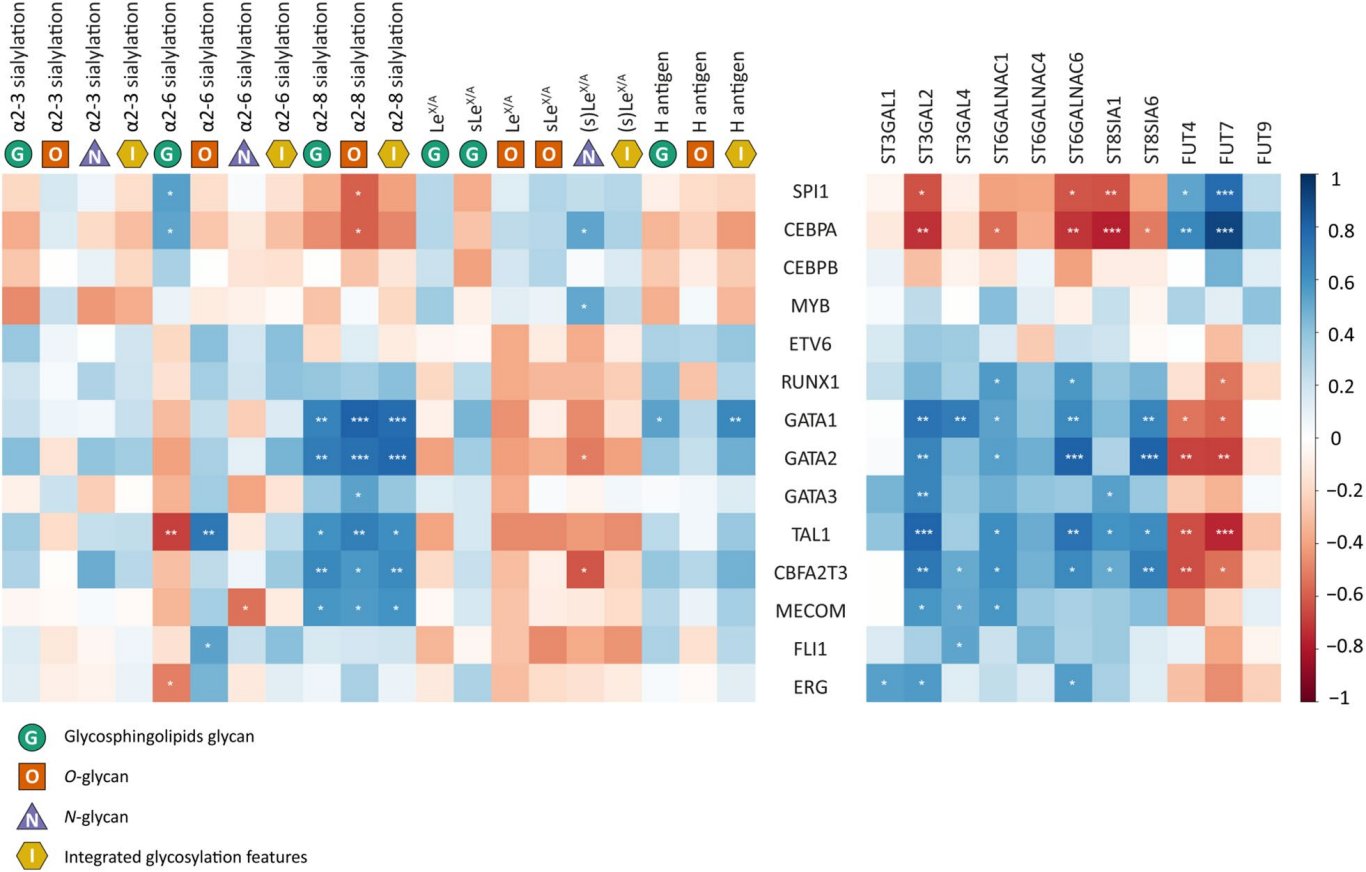
Correlation of glycosylation features of *N*-, *O*-, and GSL-glycans with the expression of selected TFs in AML cell lines. Correlation coefficients were obtained by Spearman analysis and are indicated by color as indicated in the legend. Of note, due to rather weak correlations of *ST6GALs* and glycomics data, we did not include these GSTs in our overview. Significant values are marked with * (p ≤ 0.05), ** (p ≤ 0.01,) and *** (p ≤ 0.001). Correlation coefficients and p-values are listed in the Additional file 2: Table S9

To explore the possible transcriptional regulation of glycosylation throughout all glycan classes, we correlated glycomic features and GST expression with a set of selected TFs (Additional file 2: Tables S8 and S9). Specifically, we focused on hematopoietic TFs, which are guiding normal hematopoiesis and differentiation and were shown to be specifically altered in many cases of AML and affected by mutation in specific types of AML [17, 45]. As illustrated in the correlation heatmap in Fig. 4, the (s)Le^x/a^ antigen for the integrated as well as separate glycoprotein classes showed a positive correlation with *SPI1*, *CEBPA,* and *MYB.* In line with these observations, *FUT4* and *FUT7* also exhibited positive correlations with *SPI1* and *CEBPA. FUT9* appeared to follow a similar expression pattern but did not meet the significance criteria. Interestingly, (s)Le^x/a^ expression on *N*-linked glycoproteins showed a distinct correlation compared to sLe^x/a^ expression on glycolipids. Concerning the H antigen, *GATA1* showed a significant correlation with integrated glycan classes as well as with its expression on GSL-glycans.

Intriguingly, for sialylation, depending on its linkage and involved glycan class, distinct and sometimes opposing correlations with TFs were observed. For instance, while α-2,6 sialylation on GSLs correlated with *CEBPA* and *SPI1*, an opposing correlation was observed for *N*- and *O*-glycans. Above all, highly significant positive correlations were found between α-2,8 sialylation and several TFs, a finding that was well reflected by sialyltransferase *ST8SIA1* and *ST8SIA6* expression. Interestingly, correlations of selected sialyltransferases and TFs showed a simpler picture: almost all investigated sialyltransferases exhibited significant correlation with either *SPI1*/*CEBPA* (negative correlation) or *RUNX1*/*GATAs*/*TAL1*/*CBFA2T3* (positive correlation).

### AML cell lines show distinct glycomic and transcriptomic signatures of M5 and M6 FAB classes

**Fig. 5:**
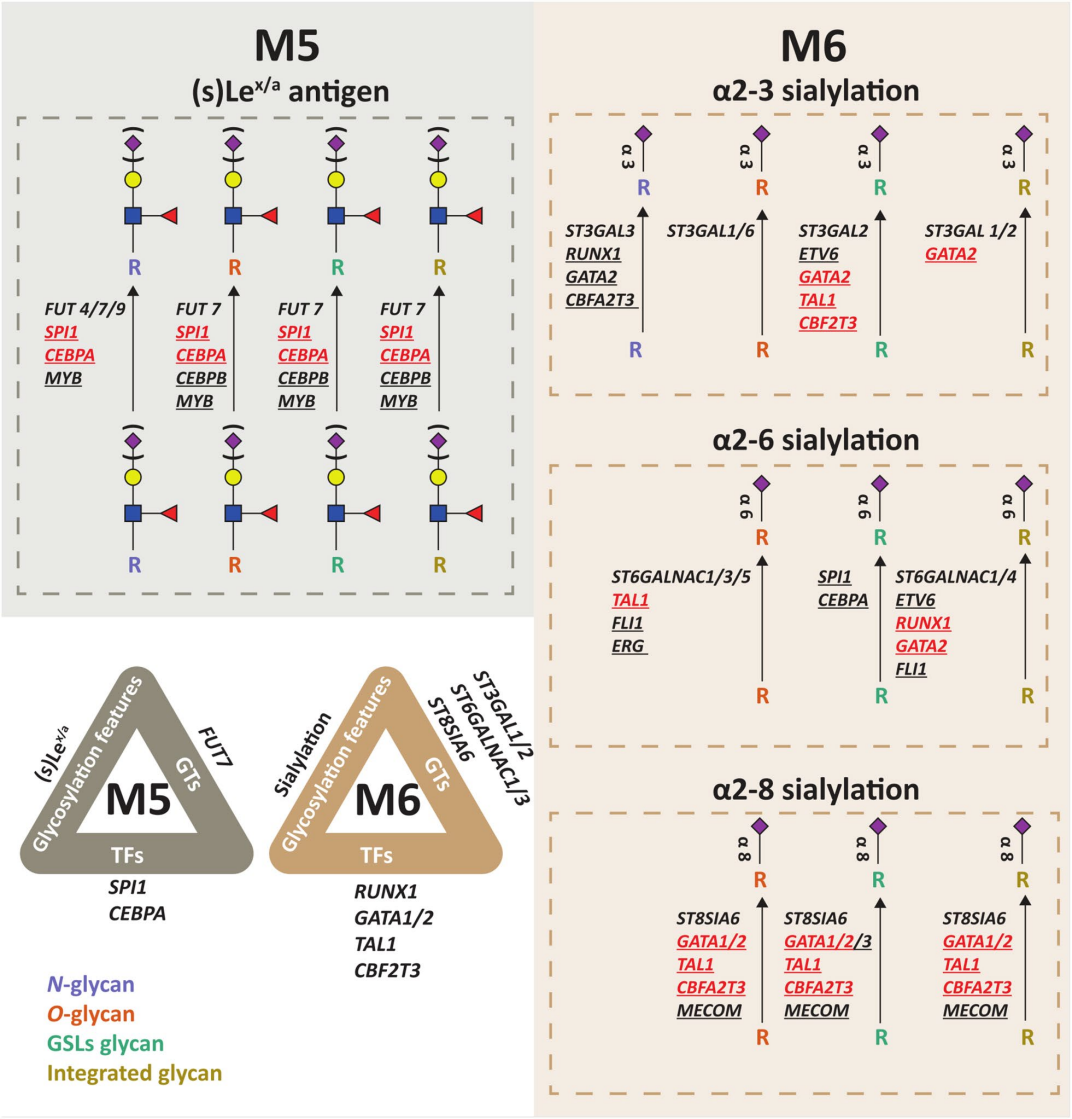
Differences in glycan signatures of M5 and M6 AML cell lines as well as corresponding GST and TF expression. M5 and M6 classes are presented as grey and brown rectangles, respectively. GSTs displayed in the figure present a positive correlation with the corresponding glycosylation feature. The underlined TFs correlate with the glycosylation features. The underlined TFs colored in red are positively correlated with GSTs

Sufficiently high numbers of cell lines belonging to either M5 or M6 were available to clearly associate FAB classification and glycomic profiles. In Fig. 5, we depict a detailed glycomic overview of these FAB classes integrating glycomics and transcriptomics data, including upstream GST and TF expression, which pinpoint the glycomic regulation of these AML subclasses resulting in their distinction. As mentioned earlier, a hallmark of the M5 subtype is its elevated (s)Le^x/a^ levels, which may be dependent mostly on *FUT7* expression and upstream *SPI1/CEBPA* expression. In contrast, the M6 subtype is characterized by especially high sialylation. To this end, *ST3GAL2*, *ST6GALNAC1/4* and *ST8SIA6* appeared to be the most correlated GSTs across all glycan classes, which in turn strongly correlated with the expression of *RUNX1*, *GATA1/2/3*, *MECOM*, *TAL1,* and *CBFA2T3*.

### GST and hematopoietic TF expression in primary AML cells

**Fig. 6:**
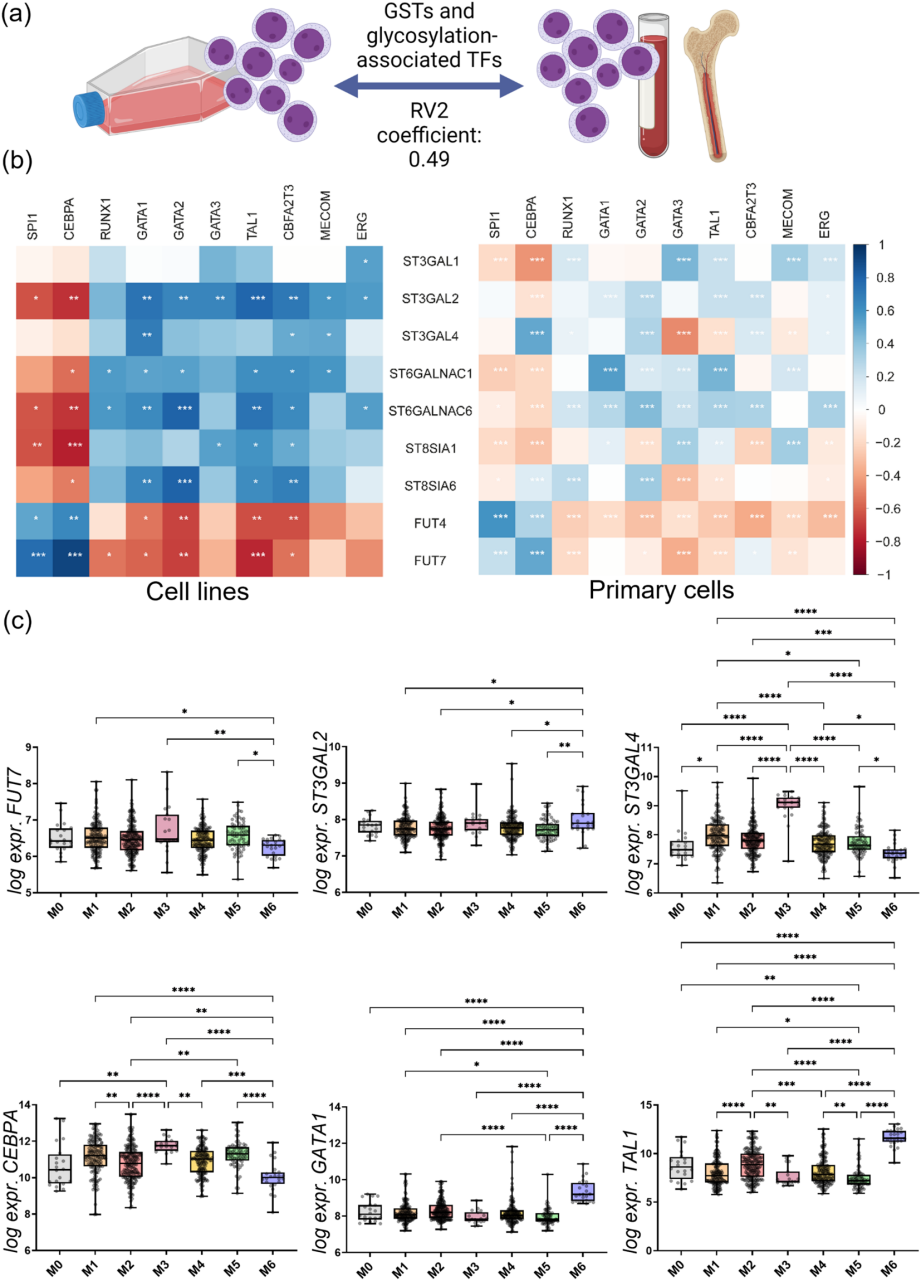
GST and TF expression in primary AML cells. **a** Determination of the matrix correlation coefficient RV2 (0.49) between expression patterns observed in cell lines and primary samples. **b** Spearman correlation of selected GSTs with TFs in AML cell lines (left) and primary AML cells (right). **c** Comparison of the expression of selected GSTs and TFs in primary AML cells grouped by FAB classification. Significances were assessed by one-way ANOVA followed by a Tukey post-hoc test. Significant values are marked with * (p ≤ 0.05), ** (p ≤ 0.01), *** (p ≤ 0.001), and **** (p ≤ 0.0001)

To explore the glyco-code of AML beyond the cell line models, we obtained transcriptomic data of AML primary cells from several previous studies and analyzed GST and TF expression as well as their associations (Additional file 2: Tables S10, S11, and S12). Based on GSTs and TFs that showed significant correlations in the cell line model (Fig. 4), we explored whether similar GST and TF correlation patterns existed in AML cell lines *versus* primary cells (Fig. 6a). After finding a good agreement as indicated by a modified RV2 coefficient [40] of 0.49, correlations between GSTs and TFs in primary cells and cell lines were visualized by heat maps (Fig. 6b). In line with our previous findings in AML cell lines, *FUT4/7* showed a significantly positive correlation with *SPI1 and CEBPA* in primary blasts. Importantly, the broad positive correlations of sialyltransferases with *RUNX1*, *GATA1/2/3*, *TAL1,* and *CBFA2T3* were also largely observed in the data obtained from primary patient material.

Prompted by these good agreements, we sought to substantiate the observation that M5 and M6 represent distinctly glycosylated AML subtypes by looking into FAB-grouped primary cell data. Moreover, by relying on a compiled dataset of 640 FAB-classified patients we were curious to see how the expression patterns in these two subtypes appear in the context of M0-4 subtypes (Fig. 6c). The M7 subclass was excluded for this analysis as only two patients appertained to this rather rare class of AML in our datasets. In total, out of our pre-defined genes of interest, we could obtain information on 14 genes from this dataset, which are depicted in Fig. 6c, Additional file 1: Fig. S1, and Additional file 2: Table S13. In clear agreement with our previous findings, *FUT7* showed high expression in M5 cells compared to M6. In accordance, *FUT4* levels were significantly higher in M5 cell lines compared to M6 (Additional file 1: Fig. S1). Moreover, both *ST6GALNAC4* and *ST3GAL2* were elevated in M6 compared to M5; a finding that fits well with the results obtained from AML cell lines. Surprisingly, *ST3GAL4*, a sialyltransferase associated with biosynthesis of the sLe^x^ epitope, was not elevated in M6 but downregulated compared to most other subtypes. Also, M3 is characterized by elevated levels of this specific sialyltransferase. Based on *ST3GAL1* expression, the M0 subclass, for which we did not obtain suitable cell line data, seems to be a FAB subtype with a rather high sialylation profile comparable to the M6 subtype (Additional file 1: Fig. S1). In addition, TF expression in primary cells was largely in agreement with the cell line models. *CEBPA*, a transcription factor highly correlated with both fucosyl- and sialyltransferases, was significantly upregulated in M5 blasts compared to M6. Again, the M3 subtype seems to resemble the phenotype of M5 also on the level of TFs. As anticipated and in contrast to *CEBPA*, the transcription factors *GATA1* and *TAL1* were highly expressed in M6. This characteristic seems to be rather unique to M6 cells compared to all other FAB classes.

## Discussion

Intrigued by the emerging role of protein and lipid-linked glycosylation in AML, we performed a meta-omics study exploring mass-spectrometric glycomics and transcriptomics data from several individual studies (Fig. 1). Taking into account both cell line models and data from primary samples we could bridge in vitro findings to the patient and explore potential implications for the clinics.

Initially, we compiled data from two recent in-depth glycomics studies focusing on a diverse panel of AML cell lines [13, 14]. After integrating the available data and grouping them into insightful glycosylation features (Additional file 2: Tables S1, S2, S3, S4, and S5), we employed PCA revealing distinct groupings of M5 and M6 cell lines (Fig. 2a). A glycomic distinction between these two FAB subtypes was indicated earlier by glycomics data obtained from proteins [13] and GSLs [14]. However, by combining the individual classes of glycosylation, the grouping of these subtypes became apparent and revealed concerted upregulation of several shared glycan traits. As evident from the PCA´s loading plot and a more detailed visualization in two radar plots (Fig. 2b, c), M5 cell lines are characterized by high levels of (s)Le^x/a^ compared to M6 cells. Surprisingly, this finding was consistently observed across all three glycan classes investigated and was mainly correlated to *FUT7* expression (Fig. 3). As sLe^x/a^ on *N*-glycoproteins [46], *O*-glycoproteins [47, 48], and GSLs [49, 50] may contribute to E-selectin binding, a concerted upregulation of sLe^x/a^- bearing biomolecules may constitute the basis for efficient molecular interactions via E-selectin.

Another prominent feature was the elevated levels of sialylation in M6 cell lines (Fig. 2). Although this observation was found for multiple sialylation-associated traits, specific traits characterized by their linkage and associated glycan class deviated markedly from this trend, e.g., α-2,6 sialylated GSLs. This demonstrates the necessity to determine this information by comprehensive glycomics experiments. Globally upregulated sialylation levels can alter immune recognition of malignant blasts [51]. Through the interaction with SIGLECs, lectin receptors expressed on many immune cells, the immune response could be modulated and in many cases dampened [52]. Interestingly, Lee et al*.* discovered that α-2,3 sialylation in the M6 cell line TF-1 is responsible for its characteristic GM-CSF-dependent growth properties [53]. Specific knockdown of either *CMAS* or *SLC35A1*, genes that are essential for sialylation, enabled its cytokine-independent growth and altered the response towards specific pathway inhibitors thereby potentially linking levels of sialylation to the optimal choice of therapy. Therefore, interfering with sialyltransferase activity may also represent an interesting novel research avenue that deserves attention in AML research.

Furthermore, M4 cell lines emerged as a distinct glycomic subtype in the PCA characterized by α-1,2 fucosylation and associated H antigen and (s)Le^b/y^ epitope expression (Fig. 2a, b). Unfortunately, to our knowledge, the role of α-1,2 fucosylated glycoproteins and glycosphingolipids is not yet explored for myeloid malignancies [54]. In other cancer entities such as breast cancer increased H2 antigen and Le^y^ expression has been linked to cancer stemness [55] and Le^y/b^ was associated with a worse prognosis [56]. Generally, α-1,2 fucosylated epitopes Le^y/b^ are known to contribute to the interaction with C-type lectin DC-SIGN expressed on dendritic cells indicating a potential role in the interactions with immune cells [57–59].

Given the fact that we found evidence for a link between AML differentiation and the glycan fingerprint, we integrated TFs in our analysis. The compiled set of hematopoietic TFs plays a key role in the pathobiology of AML and regulates its aberrantly low differentiation status [45, 60]. Considering these TFs, correlation analysis was performed with glycan traits and GST expression, respectively (Fig. 4). Several TFs were identified that correlated on the one hand with specific GSTs but were also matched by a concomitant correlation with glycomics traits on the other hand. By integrating the various obtained levels of information, we were able to get a clearer view of the difference between AML cell lines. Especially, M5 and M6 showed distinct glycomic signatures characterized by either high α-1,3/4 fucosylation or generally high sialylation profiles, respectively, (Fig. 5) suggesting that these subtypes may serve as suitable glycomic models to further study the effect of glycosylation in AML and their response to glycan-based therapeutics.

Although the potential translational value of AML cell line models was suggested earlier [61], we were excited to see that the observations gathered for AML cell lines held true for primary blasts. Because comprehensive glycomics profiles were not available for primary blasts, we explored several transcriptomics studies compiling GST/TF correlation matrices which indicated that many of the associations found in cell lines are conserved in primary blasts (Fig. 6b). Therefore, a specific expression pattern of hematopoietic TFs may regulate several important GST genes and eventually the cellular glycomic phenotype and this may not be restricted to the cell line model. *FUT7*, a potent enzyme for the synthesis of sLe^x^ [62] and a potential driver of sLe^x/a^ in AML cell lines, was positively correlated with *CEBPA*/*SPI1* in both scenarios. Interestingly, both transcription factors were shown to activate GM-CSF receptor alpha in myelomonocytic cells [63], which could be an explanation for the concerted correlation. In addition to the proposed TF-based regulation, *FUT7* may be altered epigenetically in AML [64, 65]. Another axis of regulation was represented by *GATA1/2/3* and *TAL1*: Whilst this set of TFs generally correlated negatively with *FUT7* expression, significant positive associations were found for several sialyltransferases, e.g., *ST3GAL2*, *ST6GALNAC1/4/6*, and *ST8SIA6*. In addition to the generally immune dampening functions of extensive sialylation via SIGLECs [51, 52], *ST8SIA6*-dependent α-2,8 sialylation has been recently identified as a potent immunomodulator promoting tumor growth in colorectal cancer and melanoma cell lines [66]. Moreover, α-2,8 sialylation may be interesting in the context of chemoresistance as in both cell line models as well as primary samples chemoresistant AML cells had downregulated *ST8SIA6* expression paired with upregulated *ST8SIA4* expression [67].

Prompted by the fact that the expression patterns observed for cell lines seem to largely translate to primary cells, the expression of selected GSTs and TFs was investigated across different FAB classes (Fig. 6c). As predicted by our cell line analysis, significant differences were also found between M5 and M6 subtypes in primary cells. Firstly, the investigated hematopoietic TFs clearly matched the differences found in cell lines. We assume this indicates a link to the differentiation status as classified by FAB and these TFs. Looking at TF expression patterns in combination with the TF/GST correlation maps, we already anticipated finding GST expression significantly regulated. Indeed, *FUT7* was significantly upregulated in M5 compared to M6. In combination with the upregulation *ST3GAL4* in M5, proposed to be the main sialyltransferase involved in the synthesis of sLe^x^ in myeloid leukocytes [68], this points toward a high expression of this epitope in blasts of this subtype. This may render the M5 subtype a preferential target for the use of therapeutics interfering with sLe^x^ interaction partners, such as the glycomimetic drug uproleselan [30]. In fact, high E-selectin ligand-expressing AML patients showed enhanced overall survival in a group of relapsed and refractory AML after uproleselan treatment in combination with chemotherapy [31]. Intervening with the sLe^x^—E-selectin axis may be also beneficial for eradicating minimal residual disease and relapse, which are major hurdles in the efficient treatment of AML [69]. Based on *ST3GAL4*/*FUT7* expression, M3 cells may also be a subtype with elevated sLe^x^ levels. Apart from a possible stratification of AML glyco-subtypes and their treatment, sLe^x^ may have also direct implications on the outcome of disease: in a recent report, both *ST3GAL4* and *FUT7* could be directly linked to enhanced levels of sLe^x^ on the cell surface of primary AML blasts as determined by flow cytometry [29]. More importantly, both genes were individually associated with poorer survival and a concerted upregulation of both led to the most pronounced adverse effect on mortality. Intriguingly, sLe^x^ biosynthesis may be further enhanced during chemotherapy as indicated by Ma et al*.* who found a significant upregulation in the expression of *ST3GAL4* in pairs of adriamycin sensitive and resistant AML cell lines [67]. In addition, *ST3GAL4* was significantly upregulated in patients displaying a multidrug resistance phenotype. Although not the focus of the study by Ma et al. evidence was presented that α-1,3/4 fucosylation increased in *N*-glycans obtained from adriamycin-resistant HL-60 AML cells. Precisely, the only glycan species carrying two fucoses was amongst the most significantly upregulated glycans as determined by MALDI-TOF–MS. Thus, to prevent or overcome the resistance to treatment of high sLe^x^-expressing AML subtypes such as M5, *FUT7*/*ST3GAL4* as well as their biosynthetic products may be promising targets for the development of novel glycan-targeting therapies.

*ST6GALNAC4* showed a significant upregulation in M6 cells compared to all other FAB classes investigated (Additional file 1: Fig. S1). This enzyme is a major contributor to α-2,6 sialylation of *O*-glycans, which is further corroborated by upregulation of α-2,6 sialylation on *O*-glycans in M6 cells and positive correlations in cell lines. This is of potential relevance as *ST6GALNAC4* expression was also associated with chemoresistance in AML cell lines and AML patients [67]. In addition, both *ST3GAL1/2* showed enhanced expression in M6 compared to M5 cells although *ST3GAL1* did not pass the significance threshold. This may indicate a similar scenario in the cell line model, where α-2,3 sialylation traits were clearly elevated on the glycomic level but could not be traced back to a single *ST3GAL* isoform.

Apart from M5 and M6 subtypes, we also identified significant differences in other FAB classes (Fig. 6c and Additional file 1: Fig. S1). For instance, the fairly undifferentiated M0 subtypes were characterized by high *ST3GAL1* expression paired with low *CEBPA* expression compared to other FAB subtypes. Future comprehensive glycomics assessment of primary AML will provide more detailed information regarding glycomic subtypes and the expression of specific glycans. This lack of a suitable glycomics datasets of primary blasts represents a limitation of the presented study and forced us to draw conclusions on the transcriptional level. Although, our integrated evaluation of cell lines and primary samples indicates that AML cell lines may serve as suitable in vitro surrogates to study the role of glycosylation in AML, post-transcriptional regulations could differ between these two sample groups and therefore potentially result in altered cellular glycosylation profiles. Clearly, it will be exciting to get direct glycomics-based insights and see whether our predictions based on transcriptomics data hold true. Considering AML´s considerable heterogeneity and high number of subtypes, a substantial number of patient samples is a necessity for such a glycomics experiment. Besides, we propose targeted glycoproteomics of important E-selectin ligands, e.g., ESL-1, PSGL-1, and CD44, to further broaden our understanding of the regulation and importance of the sLe^x/a^—E-selectin axis in AML.

The presented study initially investigated publicly available datasets of AML cell lines to conceive hypotheses regarding the stratification and regulation of AML glycosylation. Although this first step could have been skewed by the limited sample size of 19 cell lines, we were subsequentially validating our hypotheses on data of primary AML cells, for which several hundreds of samples were available. Regarding this validation, our hypothesis-driven approach for the analysis of primary AML represents a strength. A hypothesis-free transcriptome analysis of primary cells might have helped to reveal additional genes associated with AML glycan phenotypes, yet a biological interpretation of those findings would represent a difficult task.

In conclusion, by performing a meta-omics study relying on publicly available glycomics and transcriptomics datasets we provide exciting new insights into the glyco-code of AML. Using well-studied cell line models as a stepping stone, hypotheses on transcriptionally imprinted regulations of *N*-, *O*-, and GSL-glycosylation were conceived. Remarkably, by testing these hypotheses on primary samples a good agreement with the cell line models was found. We identified distinct glycomic subtypes in AML that associate with FAB classes M5 and M6 and are characterized by high (s)Le^x/a^ and sialylation profiles, respectively. While (s)Le^x/a^ may be governed by the concerted action of *FUT7*/*ST3GAL4* in M5, the extensive sialylation profile of M6 may be regulated amongst others by *ST3GAL1*/*2*, *ST6GALNAC4*, and *ST8SIA6*. Both glycomics data and GST expression were linked to a specific expression pattern of hematopoietic TFs that is in turn associated with certain FAB classes. These findings lay a foundation for glycomics research in AML and identify glycomic subtypes that are distinct regarding clinically relevant glycan epitopes. With the emergence of glycan-based therapies, our findings may be of significance for the glycomic stratification of the disease and may improve precision of such treatment paradigms.

## Supplementary Information

**Additional file 1**: **Figure S1. **GST and TF expression across FAB subtypes. Expression of selected GSTs and TFs was explored in primary AML blasts. Significances were assessed by one-way ANOVA followed by a Tukey post-hoc test. Significant values are marked with * (p ≤ 0.05), ** (p ≤ 0.01), *** (p ≤ 0.001), and **** (p ≤ 0.0001).

**Additional file 2**: **Table S1**. Overview of N-glycans presents in AML cell lines. **Table S2**. Overview of O-glycans presents in AML cell lines. **Table S3**. Overview of GSL-glycans presents in AML cell lines. **Table S4**. Overview of considered derived traits and corresponding values accross cell lines. **Table S5**. Quantitative values of glycosylation features of N/O- glycans and GSL glycans as well as integrated glycosylation features in each AML cell line. **Table S6**. The expression of glycosyltransferases in each AML cell line. **Table S7**. The Spearman correlation between glycosylation features and relevant glycosyltransferases in AML cell lines. **Table S8**. The expression of transcription factors in each AML cell line. **Table S9**. The Spearman correlation between transcription factors with glycosylation features and glycosyltransferases in AML cell lines. **Table S10**. The expression of glycosyltransferases in AML primary cells. **Table S11**. The expression of transcription factors in AML primary cells. **Table S12**. The Spearman correaltion between glycosyltransferases and transcription factors in AML cell lines and AML primary cells. **Table S13**. The expression of genes in AML primary cells grouped by the FAB classification.

## Data Availability

Following datasets were analyzed during the current study: GlycoPOST datasets GPST000214 (http://doi.org/10.50821/GLYCOPOST-GPST000214) and GPST000238 (http://doi.org/10.50821/GLYCOPOST-GPST000238); GEO datasets GSE122515 (https://www.ncbi.nlm.nih.gov/geo/query/acc.cgi?acc=GSE122515), GSE12417 (https://www.ncbi.nlm.nih.gov/geo/query/acc.cgi?acc=GSE12417), and GSE37642 (https://www.ncbi.nlm.nih.gov/geo/query/acc.cgi?acc=GSE37642).

## Abbreviations

AML: Acute myeloid leukemia
FAB: French-American-British
FLT3: FMS-related tyrosine kinase 3
GST: Glycosyltransferase
PCA: Principal component analysis
PMA: Phorbol 12-myristate 13-acetate
(s)Le^x/a^: (Sialyl) Lewis^x/a^
TF: Transcription factor
