# Supplementary material for: Transcriptionally imprinted glycomic signatures of acute myeloid leukemia
Source: Cell Biosci. 2023 Feb 14;13:31. doi: 10.1186/s13578-023-00981-0 (PMC9926860; doi:10.1186/s13578-023-00981-0)
Supplement: Supplementary file 1 — Additional file 1: Figure S1. GST and TF expression across FAB subtypes. Expression of selected GSTs and TFs was explored in primary AML blasts. Significances were assessed by one-way ANOVA followed by a Tukey post-hoc test. Significant values are marked with * (p ≤ 0.05), ** (p ≤ 0.01), *** (p ≤ 0.001), and **** (p ≤ 0.0001). [file 13578_2023_981_MOESM1_ESM.pdf]

Supporting Information

Transcriptionally imprinted glycomic signatures of acute myeloid leukemia

Constantin Blöchl[a,#], Di Wang[a,#], Oleg A. Mayboroda[a], Guinevere S.M. Lageveen-Kammeijer[a], and Manfred Wuhrer[a,*]

[#] *These authors contributed equally.*

[a] *Leiden University Medical Center, Center for Proteomics and Metabolomics, Albinusdreef 2, 2333 ZA Leiden, The Netherlands*

**Corresponding author: m.wuhrer@lumc.nl, tel: +31(0)71 526 6989*

TABLE OF CONTENTS

Additional file 1: Figure S1. GST and TF expression across FAB subtypes. .... page 2

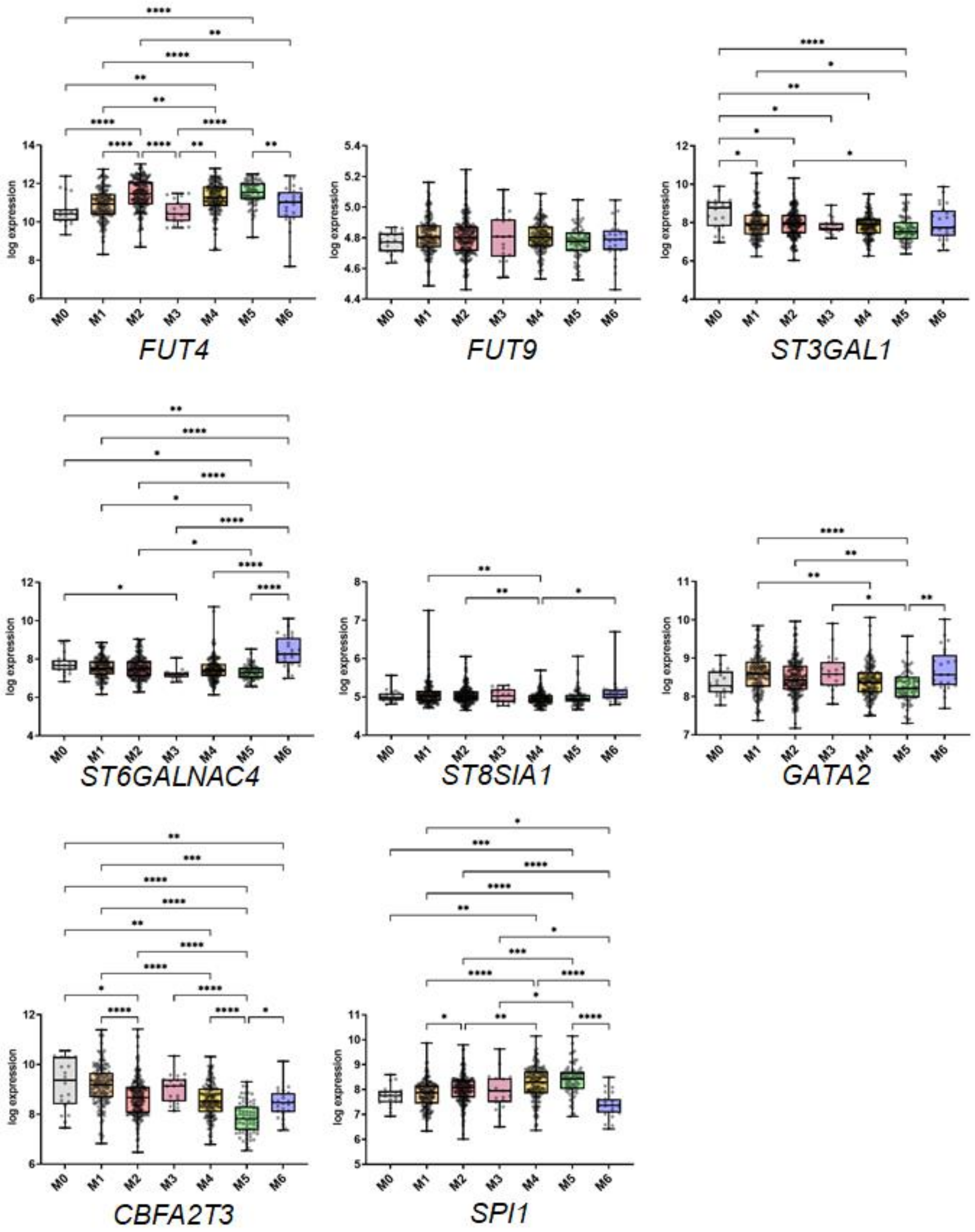

Additional file 1: Figure S1. GST and TF expression across FAB subtypes. *Expression of selected GSTs and TFs was explored in primary AML blasts. Significances were assessed by one-way ANOVA followed by a Tukey post-hoc test. Significant values are marked with * (p ≤ 0.05), ** (p ≤ 0.01), *** (p ≤ 0.001), and **** (p ≤ 0.0001).*
